# Seroprevalence of SARS-CoV-2 in Brazil: A systematic review and meta-analysis

**DOI:** 10.1016/j.clinsp.2023.100233

**Published:** 2023-06-13

**Authors:** Gerusa Maria Figueiredo, Fátima Mitiko Tengan, Sergio Roberto Campos, Expedito José Luna

**Affiliations:** a Departamento de Medicina Preventiva da Faculdade de Medicina da Universidade de São Paulo, São Paulo, SP, Brazil; b Departamento de Moléstias Infecciosas e Parasitarias da Faculdade de Medicina da Universidade de São Paulo, São Paulo, SP, Brazil

**Keywords:** Seroprevalence, SARS-CoV-2, Systematic review, Meta-analysis

## Abstract

**Objectives:**

To summarize the data on SARS-CoV-2 seroprevalence surveys conducted in Brazil before the introduction of vaccines

**Methods:**

The authors conducted a systematic review and meta-analysis on the seroprevalence of SARS-CoV-2 infection in Brazil. The present review followed the PRISMA guidelines. The authors searched Medline, Embase, and LILACS databases for serologic surveys conducted in the Brazilian population, in the period from 01/10/2019 to 07/11/2021, without language restrictions. The authors included studies that presented data concerning SARS-CoV-2 antibodies seroprevalence in Brazil and had a sample size ≥50 individuals. Considering the expected heterogeneity between studies, all analyses were performed using the random effects model, and heterogeneity was assessed using the I^2^ statistic

**Results:**

Of 586 publications identified in the initial searches, 54 were included in the review and meta-analysis, which contained the results of 135 surveys, with 336,620 participants. The estimated seroprevalence was 11.0%, ranging from 1.0% to 83.0%, with a substantial heterogeneity (I^2^ = 99.55%). In subgroup analyses, the authors observed that the prevalence of SARS-CoV-2 antibodies was 13.0% in blood donors, 9.0% in the population-based surveys, 13% in schoolchildren, and 11.0% in healthcare workers.

**Conclusions:**

Seroprevalence increases over time. Large differences were observed among the regions of the country. It was higher in the Northern region, decreasing towards the South. The present results may contribute to the analysis of the spread of SARS-CoV-2 infection in the Brazilian population before vaccination, one of the factors that may be influencing the clinical presentation of COVID-19 cases related to the new variants, as well as the effectiveness of the vaccination program.

## Highlights

• The seroprevalence of SARS-CoV-2 antibodies in Brazil in 2020 was 11%.
• Seroprevalence increased with time, 1% in the first and 83% in the last quarter of the year.
• Seroprevalence was higher in the Northern region, decreasing as one moves south.

## Introduction

The COVID-19 pandemic, caused by SARS-CoV-2 was first reported in Wuhan, China, in December 2019 [1]. It quickly spread globally and constitutes the largest pandemic of the last 100 years. In Brazil, the first case of SARS-CoV-2 was confirmed in late February 2020. In the beginning, transmission was restricted to a few large cities where imported cases were detected, and local transmission was established. In late March and April, the disease spread from these original entry points to the whole country. Serologic surveillance is one of the recommended strategies to monitor the spread of SARS-CoV-2 infection in the population, once asymptomatic and moderate cases may be underreported. Serologic surveys provide additional information regarding the spread of SARS-CoV-2 infection in the population and help to understand the spread of infection in the population and their immunity. This knowledge was of great importance in that period, when vaccine trials were still being carried out and real manufacturing and distribution capacity throughout the world were not in place, and just non-pharmacological measures for prevention and control were available.

In April 2020, serological surveys were started for this purpose. A large national seroprevalence survey was undertaken in Brazil, and several others with restricted geographical coverage or convenience samples were carried out. Until December 2020, several studies were carried out with highly variable estimates of seroprevalence that could largely be due to differences in attack rates, but which also feature heterogeneous sampling strategies and assays used.

So far, there is no study summarizing these surveys in Brazil and so the authors conducted a systematic review and meta-analysis with this objective.

The results of the present study may contribute to the analysis of the spread of SARS-CoV-2 infection in the Brazilian population before vaccination, one of the factors that may be influencing the clinical presentation of COVID-19 cases related to the new variants, as well as the effectiveness of the vaccination program.

## Methods

The authors conducted a systematic review of published articles and a manual search, on the seroprevalence of SARS-CoV-2 infection in Brazil. The present review was conducted and reported in accordance with the Preferred Reporting Items for Systematic Reviews and Meta-Analyses (PRISMA) Statement [2].

### Search strategies

The search was carried out in Medline (through the PubMed platform), Embase and Latin American Literature (Lilacs) databases, without language restrictions.

In Medline, the terms COVID, COVID-19, COV, coronavir*, Sars, SARS-CoV-2, 2019-nCoV, prevalence, cross-sectional study, seroepidemiology, serosurvey, serology, serological survey and Brazil, were used, restricting the surveys to humans and in the period from 01/10/2019 to 07/11/2021 (more details on the search strategy in the Supplementary File 1).

In Embase, the terms *'coronavirus disease 2019′:ti,ab,kw AND (prevalence:ti,ab,kw OR seroprevalence:ti,ab,kw) AND brazil:ti,ab,kw, were used,* no time or human restrictions.

In Lilacs, the authors used the terms *(“SARS-CoV-2”) or “COVID-19” [Descritor de assunto] and “BRASIL” [Descritor de assunto],* no time or human restrictions.

The surveys were carried out with different strategies, such as seroprevalence on representative samples of the population, the country as a whole, states, municipalities, or regions, seroprevalence surveys carried out on samples of specific population groups, and surveys on convenience samples.

Through a manual search of references in selected articles and review articles on the topic, the authors sought to identify other relevant studies missed in the searches. The authors also investigated the websites of Municipal and State Health Departments in search of official reports and, considering that the topic of the review is recent and that many researchers are dedicating themselves to its research, the authors also added articles available to the public, but not reviewed by peers (sites: MedRxiv, BioRxiv, Euro PMC Preprint, BMC, SSRN, Wellcome Open Search). Two authors (SRCC and FT) selected articles, examining titles and abstracts, resulting in a list of potentially relevant sources. After reading the full text of the selected references, the articles were selected for inclusion in the review. Disagreements were resolved by discussion and consensus. Study authors were contacted when data were not clear enough.

### Selection of studies

Only articles/documents that contained original data on the seroprevalence of SARS-CoV-2 infection in Brazil carried out in 2020 and whose sample size was greater than or equal to 50, were included. The authors did not include case reports, case series, review articles, comments, studies whose participants did not live in Brazil, or articles that contained the same data. Regarding the latter studies, the article with the most complete data was included in the present review.

The following definition for SARS-CoV-2 infection was used: the presence of anti-SARS-CoV-2 antibodies IgG and/or IgM to SARS-CoV-2 measured by Enzyme Immunoassay (ELISA) or Chemiluminescent Immunoassay (CLIA test) or rapid tests serological Immunochromatography (ICA).

### Data extraction

Two investigators (SRCC, FMT) collected data independently and disagreements were resolved through discussions and consensus. The following data were collected: name of the first author/document title, State of Brazil where the study was carried out, data collection period, sample size, gender, age, race, number of positive individuals for anti-SARS-CoV-2 and diagnostic method for detecting anti-SARS-CoV-2.

Inclusion criteria: Seroprevalence surveys were conducted in Brazil, with a sample ≥50, without other restrictions.

Exclusion criteria: Reports of clinical trials of therapeutic or preventive products, studies without one of the following data: number of participants; the number of participants with reagent results for SARS-CoV-2 antibodies; studies that did not explicitly state their geographic scope; studies that did not explicitly state the laboratory assay that was used for antibodies detection; studies that did not explicitly state the methods for sample selection.

### Statistical analysis

Considering the expected heterogeneity between studies, all meta-analyses were performed using the random effects model, which includes variation among studies. Heterogeneity was assessed using the I^2^ statistic, which describes the percentage of variation among studies that is due more to heterogeneity than to chance [3]. I^2^ values greater than 25%, 50%, and 75% are considered evidence of mild, moderate to high heterogeneity among studies. Low values of I^2^ suggest that variability among estimates is compatible with random variation.

To investigate possible causes of heterogeneity among studies, the authors performed a meta-analysis of the following subgroups.

1. Study groups: The surveys were grouped into the following subgroups: population-based surveys with randomly selected samples, blood donors, schoolchildren, and healthcare workers. The surveys addressing other population groups, such as indigenous people, pregnant women, patients with different chronic conditions, self-selected samples, and others, were included just in the main seroprevalence meta-analysis. The authors decided not to compose other subgroups due to the small number of surveys in each category.
2. Studies carried out by trimester (in the 1st, 2nd, 3rd, and 4th trimester).
3. Studies carried out in each region of Brazil.

Potential sources of heterogeneity were also investigated by regression analysis. The objective of which was to report differences in the size of the effect of the study characteristics. The following factors were examined: study group (population-based or not), sample size (continuous variable), and laboratory method for detecting anti-SARS-CoV-2 (rapid test or not.).

To examine the publication bias, the authors used tests proposed by Begg and Mazumdar [4] and Egger et al [5].

The authors performed four sensitivity analyses, considering only studies with:

1. sample size < 100;
2. sample size < 500;
3. Sample size < 1000;
4. Studies published in scientific journals;
5. Studies that used rapid tests (immunochromatography) to detect anti-SARS-CoV-2 antibodies.

## Results

**Fig. 1:**
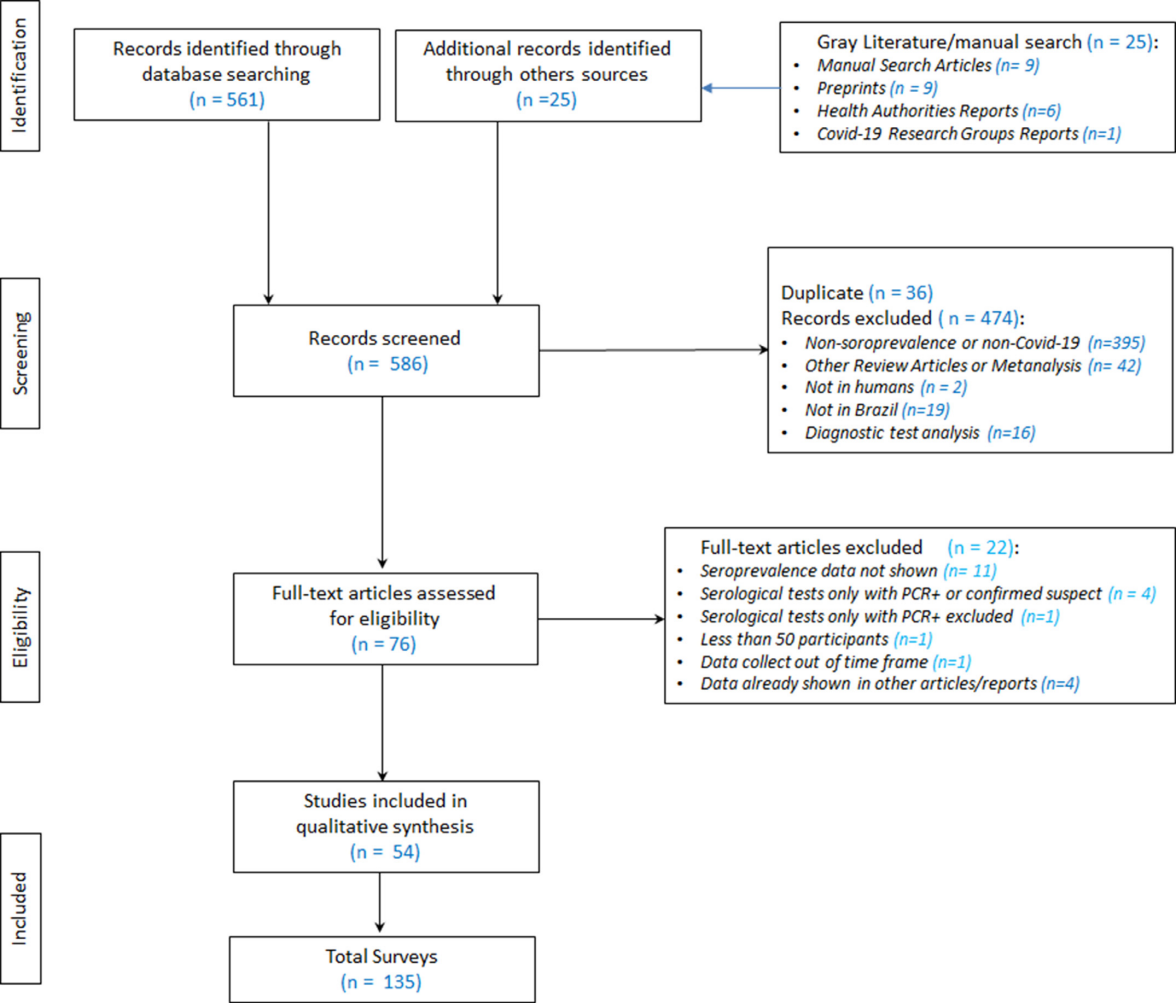
flowchart of the identification, inclusion, and exclusion of studies.

The authors initially identified 586 publications in the databases (MEDLINE, Lilacs and Embase), and in manual searching (Fig. 1 Supplementary file 1) After the exclusion of duplicates (36), the authors analyzed 550 references by reading the abstracts. 474 were subsequently excluded, leaving 76 references selected for full-text reading. After reading the full text of the 76 articles, the authors ultimately selected 54 for final inclusion in the review.

Through the search and selection of articles and/or reports on the prevalence of SARS-CoV-2 infection in Brazil shown in Fig. 1 (Search and Selection Flowchart), the authors identified 54 relevant reference sources for this review, in which 135 serological surveys were identified, with a total of 336,62  participants, about the topic: 7 articles/reports containing two surveys each; [6], [7], [8], [9], [10], [11], [12] two articles/reports containing three surveys each [13,14], one report with four surveys [15], two articles containing five surveys each [16,17], two articles/reports containing seven surveys each [18,19], three articles/reports containing eight surveys each [20], [21], [22], one report with data from ten surveys [23] and one article with information from 18 surveys [24].

### General characteristics of selected surveys

The general characteristics of the selected surveys are shown in Table 1. Of the 135 surveys, 91 (67.4%) were published in the year 2020 and 44 (32.6%) in 2021. Three surveys and studies (2.2%) were carried out in the Central-West region of Brazil, 14 (10.4%) in the North region, 35 (25.9%) in the Northeast region, 15 (11.1%) in the South region and 68 (50.4%) in the Southeast. Data from 5 (3.7%) surveys were collected in the first quarter of 2020, 62 (45.9%) in the second quarter, 46 (34.0%) in the third quarter, 13 (9.6%) in the fourth trimester, and the rest of the survey studies [9] were carried out in more than one quarter, as shown in Table 1. The most frequent surveys were population-based studies (58.5%), in blood donors (14.8%), schoolchildren (4.4%), and health workers (3.7%). The most frequently used diagnostic test for the detection of anti-SARS-CoV-2 was immunochromatography (70.4%), followed by ELISA (22.4%) and CLIA (7.4%).

**Table 1:** Estimation of the prevalence of SARS-CoV-2 in studies conducted in Brazil.

**Author**	**Publication Year**	**Type of Participants**	**Trimester**	**State**	**Region**	**Diagnostic Test**	**Total**	**Positives**	**%Male**	**%White**
Barros ENC et al. [25].	2021	Care Facilities Patients and Workers	2	SP	SE	ICA	209	24	35.4	74.2
Caramelli B et al. [26].	2021	Sport and Social Club Members	2	SP	SE	ELISA	938	54	47.0	
Cleto-Yamane TL et al. [27].	2021	Immunosuppressed Patients	2,3	RJ	SE	ICA	114	35		
Costa SF et al. [28].	2021	Healthcare Workers	2	SP	SE	ICA	4987	701	27.1	64.3
Diegoli H et al. [6].	2021	Population Based Randomly Selected	3	SC	S	ICA	3245	187		
Diegoli H et al. [6].	2021	Population Based Randomly Selected	2	SC	S	ICA	1158	26		
Garibaldi PMM et al. [29].	2021	Outbreak investigation in nursing facility	2	SP	SE	ICA	49	24		
Pontes GS et al. [30].	2021	Indigenous People	4	AM	N	ELISA	280	170	42.1	
Gurgel RQ et al. [31].	2021	Asymptomatic Patients in Hospital	1	SE	NE	ICA	987	16		
Silva HP et al. [32].	2021	Indigenous People	4	PA	N	ELISA	101	84	42.6	
Miraglia JL et al. [33].	2021	Population Based Randomly Selected	4	SP	SE	ICA	272	119	33.5	
Chiste JA et al. [34].	2021	Pregnant Women	3,4	PR	S	ICA	195	17		
Lalwani P et al. [35].	2021	Self-Selected [by media]	3	AM	N	ELISA	3046	886	39.1	
Trafane LF et al. [36].	2021	Sickle cell disease patients	3,4	SP	SE	CLIA	135	15	57.0	
Nicolette VC et al. [37].	2021	Population Based Randomly Selected	4	AC	N	ELISA	1281	448	54.0	
Oliveira MS et al. [38].	2021	Healthcare Workers	1,2,3	SP	SE	ELISA	1996	110	29.0	
Pasqualotto AC et al. [39].	2021	Military Forces	3	RS	S	ELISA	1592	28		
Rodrigues EPS et al. [40].	2021	Indigenous People	3	PA	N	ELISA	100	73	51.0	
Santana FM et al. [41].	2021	Immunosuppressed Patients	1,2,3	SP	SE	CLIA	100	21	15.0	
Araujo AAS et al. [42].	2021	University Students	3	SE	NE	ICA	276	62		
Araujo AAS et al. [43].	2021	Population Based Randomly Selected	3	SE	NE	ICA	5615	652	40.3	
Martinez EZ et al. [7].	2021	Population Based Randomly Selected	2	SP	SE	ICA	646	19		
Martinez EZ et al. [7].	2021	Population Based Randomly Selected	2	SP	SE	ICA	709	9	43.6	63.9
Tess BH et al. [44].	2021	Population Based Randomly Selected	2	SP	SE	CLIA	463	30		65.2
Maciel ELN et al. [45].	2021	Population Based Randomly Selected	2	ES	SE	ICA	1447	161	35.0	35.6
Pinto Junior VC et al. [13].	2021	Population Based Randomly Selected	4	CE	NE	ICA	423	107		
Pinto Junior VC et al. [13].	2021	Population Based Randomly Selected	4	CE	NE	ICA	854	250		
Pinto Junior VC et al. [13].	2021	Population Based Randomly Selected	4	CE	NE	ICA	282	59		
Lugon P et al. [8].	2021	Favela Children	2,3	RJ	SE	CLIA	242	79		
Lugon P et al. [8].	2021	Favela Children Contacts	2,3	RJ	SE	CLIA	231	72		
Albuquerque JOM et al. [20].	2021	Population Based Randomly Selected	2	SP	SE	ICA	2645	247		
Albuquerque JOM et al. [20].	2021	Population Based Randomly Selected	3	SP	SE	ICA	2481	261	36.0	52.1
Albuquerque JOM et al. [20].	2021	Population Based Randomly Selected	3	SP	SE	ICA	2323	282		
Albuquerque JOM et al. [20].	2021	Population Based Randomly Selected	3	SP	SE	ICA	2529	296		
Albuquerque JOM et al. [20].	2021	Population Based Randomly Selected	3	SP	SE	ICA	2447	298	36.0	51.6
Albuquerque JOM et al. [20].	2021	Population Based Randomly Selected	3	SP	SE	ICA	2225	303		
Albuquerque JOM et al. [20].	2021	Population Based Randomly Selected	3	SP	SE	ICA	2125	270		
Albuquerque JOM et al. [20].	2021	Population Based Randomly Selected	3	SP	SE	ICA	2012	244	34.9	49.8
Couto AC et al. [9].	2021	Shelter homeless people	3	SP	SE	ELISA	203	111	88.7	29.6
Couto AC et al. [9].	2021	Shelter Workers	3	SP	SE	ELISA	87	43	50.6	34.5
Cristelli MP et al. [46].	2021	Kidney Transplant Recipients	3	SP	SE	ICA	416	34	59.1	45.0
Bernardes-Souza B et al. [10].	2021	Population Based Randomly Selected	2	MG	SE	ICA	400	2	39.0	52.8
Bernardes-Souza B et al. [10].	2021	Population Based Randomly Selected	2	MG	SE	ICA	400	7	49.0	58.8
Stringari LL et al. [47].	2021	Blood Donors	1,2	ES	SE	CLIA	7370	210	32.5	
Amorim Filho L et al. [48].	2020	Blood Donors	2	RJ	SE	ICA	2857	114		
Batista KBC et al. [49].	2020	Population Based Randomly Selected	2	SP	SE	ICA	2.342	33	46.5	
Borges LP et al. [50].	2020	Firefighters	2	SE	NE	ICA	2635	218		
Buss LF et al. [24].	2020	Blood Donors	3	AM	N	CLIA	881	242		
Buss LF et al. [24].	2020	Blood Donors	3	AM	N	CLIA	1147	419		
Buss LF et al. [24].	2020	Blood Donors	4	AM	N	CLIA	882	183		
Buss LF et al. [24].	2020	Blood Donors	3	AM	N	CLIA	868	214		
Buss LF et al. [24].	2020	Blood Donors	2	AM	N	CLIA	829	46		
Buss LF et al. [24].	2020	Blood Donors	1	AM	N	CLIA	821	1		
Buss LF et al. [24].	2020	Blood Donors	2	AM	N	CLIA	911	422		
Buss LF et al. [24].	2020	Blood Donors	2	AM	N	CLIA	901	359		
Buss LF et al. [24].	2020	Blood Donors	1	AM	N	CLIA	832	6		
Buss LF et al. [24].	2020	Blood Donors	3	SP	SE	CLIA	906	113		
Buss LF et al. [24].	2020	Blood Donors	3	SP	SE	CLIA	879	84		
Buss LF et al. [24].	2020	Blood Donors	4	SP	SE	CLIA	877	100		
Buss LF et al. [24].	2020	Blood Donors	3	SP	SE	CLIA	933	101		
Buss LF et al. [24].	2020	Blood Donors	2	SP	SE	CLIA	900	27		
Buss LF et al. [24].	2020	Blood Donors	1	SP	SE	CLIA	799	7		
Buss LF et al. [24].	2020	Blood Donors	2	SP	SE	CLIA	880	105		
Buss LF et al. [24].	2020	Blood Donors	2	SP	SE	CLIA	826	44		
Buss LF et al. [24].	2020	Blood Donors	1	SP	SE	CLIA	2454	22		
Costa SF et al. [51].	2020	Healthcare Workers	2	SP	SE	CLIA	4417	528		
Espírito Santo. SES [21]	2020	Elementary or High School Students	4	ES	SE	ICA	3062	340	48.9	33.6
Espírito Santo. SES [21]	2020	Educational Professionals	4	ES	SE	ICA	3922	304	27.5	43.5
Espírito Santo. SES [21]	2020	Population Based Randomly Selected	2	ES	SE	ICA	4612	97		
Espírito Santo. SES [21]	2020	Population Based Randomly Selected	3	ES	SE	ICA	7831	511		
Espírito Santo. SES [21]	2020	Population Based Randomly Selected	2	ES	SE	ICA	4644	239		37.9
Espírito Santo. SES [21]	2020	Population Based Randomly Selected	3	ES	SE	ICA	7678	517		
Espírito Santo. SES [21]	2020	Population Based Randomly Selected	2	ES	SE	ICA	4633	341		36.0
Espírito Santo. SES [21]	2020	Population Based Randomly Selected	2	ES	SE	ICA	4922	473		35.1
Gomes CC et al. [52].	2020	Population Based Randomly Selected	2	ES	SE	ICA	4.608	97	39.0	38.3
Horta BL et al. [16].	2020	Population Based Randomly Selected	2		CO	ICA	9792	43		
Horta BL et al. [16].	2020	Population Based Randomly Selected	2		SE	ICA	21,860	149		
Horta BL et al. [16].	2020	Population Based Randomly Selected	2		S	ICA	14,888	31		
Horta BL et al. [16].	2020	Population Based Randomly Selected	2		NE	ICA	26,809	776		23.6
Horta BL et al. [16].	2020	Population Based Randomly Selected	2		N	ICA	16,013	1065		19.3
Ismael C et al. [53].	2020	Healthcare Workers	2	RJ	SE	ICA	60	3		
Silva AAM et al. [54].	2020	Population Based Randomly Selected	3	MA	NE	CLIA	3156	1167	38.0	
Paula CC et al. [11].	2020	Self-Selected	2	MT	CO	ICA	2.144	252		
Paula CC et al. [11].	2020	Self-Selected	3	MT	CO	ICA	4.248	1161		
Picon RV et al. [12].	2020	Population Based Randomly Selected	2	RS	S	ICA	1450	40	34.0	79.3
Picon RV et al. [12].	2020	Population Based Randomly Selected	2	RS	S	ICA	1127	20	35.8	100.0
Teresina. FMS [23]	2020	Population Based Randomly Selected	2	PI	NE	ICA	900	98	47.0	
Teresina. FMS [23]	2020	Population Based Randomly Selected	2	PI	NE	ICA	900	139	47.0	
Teresina. FMS [23]	2020	Population Based Randomly Selected	2	PI	NE	ICA	900	163	47.0	
Teresina. FMS [23]	2020	Population Based Randomly Selected	2	PI	NE	ICA	900	174	47.0	
Teresina. FMS [23]	2020	Population Based Randomly Selected	3	PI	NE	ICA	900	163	47.0	
Teresina. FMS [23]	2020	Population Based Randomly Selected	3	PI	NE	ICA	900	226	47.0	
Teresina. FMS [23]	2020	Population Based Randomly Selected	3	PI	NE	ICA	900	210	47.0	
Teresina. FMS [23]	2020	Population Based Randomly Selected	3	PI	NE	ICA	900	206	47.0	
Teresina. FMS [23]	2020	Population Based Randomly Selected	3	PI	NE	ICA	900	190	47.0	
Teresina. FMS [23]	2020	Population Based Randomly Selected	3	PI	NE	ICA	900	180	47.0	
Rio de Janeiro. SMS [17]	2020	Population Based Randomly Selected	2	RJ	SE	ICA	3211	556	31.1	24.9
Rio de Janeiro. SMS [17]	2020	Population Based Randomly Selected	2	RJ	SE	ICA	3202	396	28.5	26.9
Rio de Janeiro. SMS [17]	2020	Population Based Randomly Selected	2	RJ	SE	ICA	3200	300	31.3	26.0
Rio de Janeiro. SMS[17]	2020	Population Based Randomly Selected	3	RJ	SE	ICA	3170	319	28.5	27.3
Rio de Janeiro. SMS [17]	2020	Population Based Randomly Selected	3	RJ	SE	ICA	3048	233	31.8	29.8
Hallal PC et al. [22].	2020	Population Based Randomly Selected	2	RS	S	ICA	4500	2		
Hallal PC et al. [22].	2020	Population Based Randomly Selected	2	RS	S	ICA	4500	6		
Hallal PC et al. [22].	2020	Population Based Randomly Selected	2	RS	S	ICA	4500	10		
Hallal PC et al. [22].	2020	Population Based Randomly Selected	2	RS	S	ICA	4500	8		
Hallal PC et al. [22].	2020	Population Based Randomly Selected	2	RS	S	ICA	4500	21		
Hallal PC et al. [22].	2020	Population Based Randomly Selected	3	RS	S	ICA	4500	43		
Hallal PC et al. [22].	2020	Population Based Randomly Selected	3	RS	S	ICA	4500	55		
Hallal PC et al. [22].	2020	Population Based Randomly Selected	3	RS	S	ICA	4500	62		
Sales MJT et al. [55].	2020	Population Based Randomly Selected	2	PE	NE	ICA	904	39		
Melo MS et al. [56].	2020	Healthcare Workers	2	SE	NE	ICA	471	101		
Oliveira LMS et al. [57].	2020	Outpatients in Public Hospital	3	SP	SE	CLIA	439	61	35.5	
Silva VO et al. [58].	2020	Healthcare Workers	2,3	SP	SE	ICA	406	35	27.1	
São Paulo. SMS [15]	2020	Elementary or High School Students	3	SP	SE	ICA	2659	428		36.3
São Paulo. SMS [15]	2020	Elementary or High School Students	3	SP	SE	ICA	2518	460		
São Paulo. SMS [15]	2020	Elementary or High School Students	3	SP	SE	ICA	2182	360		
São Paulo. SMS [15]	2020	Elementary or High School Students	3	SP	SE	ICA	2069	331		
Campinas. SMS [59]	2020	Population Based Randomly Selected	2	SP	SE	ICA	1937	43	41.4	
SoroEpi-MSP [14]	2020	Population Based Randomly Selected	2	SP	SE	CLIA	1183	135	46.6	
SoroEpi-MSP [14]	2020	Population Based Randomly Selected	3	SP	SE	CLIA	1470	127		
SoroEpi-MSP [14]	2020	Population Based Randomly Selected	4	SP	SE	CLIA	1129	296	46.6	
Vieira MACS et al. [18].	2020	Population Based Randomly Selected	2	PI	NE	ICA	900	5		
Vieira MACS et al. [18].	2020	Population Based Randomly Selected	2	PI	NE	ICA	900	8		
Vieira MACS et al. [18].	2020	Population Based Randomly Selected	2	PI	NE	ICA	900	13		
Vieira MACS et al. [18].	2020	Population Based Randomly Selected	2	PI	NE	ICA	900	18		
Vieira MACS et al. [18].	2020	Population Based Randomly Selected	2	PI	NE	ICA	900	34		
Vieira MACS et al. [18].	2020	Population Based Randomly Selected	2	PI	NE	ICA	900	52		
Vieira MACS et al. [18].	2020	Population Based Randomly Selected	2	PI	NE	ICA	900	79		
Ceara. SES [19]	2020	Population Based Randomly Selected	2	CE	NE	ICA	3301	468	34.5	24.1
Ceara. SES [19]	2020	Population Based Randomly Selected	3	CE	NE	ICA	3306	433	33.6	25.8
Ceara. SES [19]	2020	Elementary or High School Students	3	CE	NE	ICA	3327	88	49.6	27.1
Ceara. SES [19]	2020	Population Based Randomly Selected	4	CE	NE	ICA	3331	485	30.3	24.2
Ceara. SES [19]	2020	Population Based Randomly Selected	2	CE	NE	ICA	800	92	39.6	19.5
Ceara. SES [19]	2020	Population Based Randomly Selected	3	CE	NE	ICA	485	64	34.0	26.0
Ceara. SES [19]	2020	Population Based Randomly Selected	2	CE	NE	ICA	700	11	35.3	32.0

Notes: ICA, Immunocromatography Assays; ELISA, Enzyme-Linked Immunosorbent Assay; CLIA, Chemiluminescence Immunoassay.

### Meta-analysis

a) General: The authors found a general estimated prevalence of 11.0% (95% CI 11.0‒12.0), ranging from 1.0% (95% CI 0.0‒1.0) to 83.0% (95% CI 75.0‒89.0), with substantial heterogeneity (I^2^ = 99.55%) (Supplementary File 2).
b) Subgroups:
i. In population-based surveys (Table 2) the estimated prevalence was 9.0% (95% CI 6.0%‒9.0%), in blood donors it was 13.0% (95% CI 11.0‒13.0), in schoolchildren it was 13.0% (95% CI 7.0‒20.0) and in health workers it was 11.0% (95% CI 7.0‒15.0) (more details in the Supplementary File 3, 4, 5, 6).

**Table 2:** Seroprevalence of anti-SARS-CoV-2 antibodies in Brazil, in selected subgroup [9]..

**Subgroup**	**Number of Surveys**	**Seroprevalence% (95% CI)**
Population based	79	9.0 (95% CI 6.0‒9.0)
Blood donors	20	13.0 (95% CI 11.0‒16.0)
Schoolchildren	6	13.0 (95% CI 7.0‒20.0)
Healthcare workers	6	11.0 (95% CI 7.0‒15.0)
Trimester (2020)		
1	5	1.0 (95% CI 0.00‒0.01)
2	62	7.0 (95% CI 6.0‒7.0)
3	46	73.0 (95% CI 64.0‒81.0)
4	13	83.0 (95% CI 75.0‒89.0)
Region of Brazil		
North	15	29.0 (95% CI 24.0‒35.0)
Northeast	35	14.0 (95% CI 12.0‒16.0)
Central-West	3	13.0 (95% CI 3.0‒30.0)
Southeast	67	12.0 (95% CI 11.0‒13.0)
South	15	1.0 (95% CI 1.0‒1.0)
ii. Yet, in Table 2, the authors can see the estimates of anti-SARS-CoV-2 prevalence, separating the surveys according to the period (a quarter of 2020) in which the data were collected. The highest prevalence was observed in the 3rd and 4th trimesters (73.0% and 83.0%, respectively).
iii. Finally, analyzing by regions of Brazil, the North region had the highest prevalence rate (Table 2) 32.0% (followed by the Northeast (13.0%), Central-Western (13.0%), Southeast (12.0%) and, finally, the South region (1.0%).
c) Sensitivity analysis:
iv. Excluding surveys with sample sizes ≤ 100, ≤ 500 and ≤ 1000, the prevalence estimates found was 11.0% (95% CI 11.0‒12.0), 10.0% (9.0‒12.0), 11.0% and 9.0% (9.0‒10.0), respectively. Supplementary File 7, 8, 9).
v. The estimated prevalence of published and peer-reviewed surveys was 10.0% (10.0–11.0) Supplementary File 10.
lowerRoman%1 When analyzing just the surveys that used the rapid test to detect the anti-SARS-CoV-2 antibody (immunochromatography), the authors observed a prevalence of 9.0% (95% CI 9.0‒10.0). Supplementary File 11.
d) Others analysis:
vi. The estimated prevalence of -SARS-CoV-2 antibodies in male participants was 18.0% (95% CI 17.0‒20.0) and 22.0% (95% CI 20.0‒25.0) in females; in white participants it was 8.0% (7.0‒9.0) and in non-white participants, it was 11.0% (9.0‒13.0). It should be noted that the number of articles that presented these variables was small.
vii. Meta-regression: The authors tested the variables “sample size” (continuous variable), rapid test or not, population-based study or not, and whether published after peer review or not. The first 3 variables showed a significant contribution to the outcome (Table 3).

**Table 3:** Multivariate meta-regression analysis of the anti-SARS-CoV-2 seroprevalence studies in Brazil.

	**Meta regression coeficient**	**p-value**	**95% (CI)**
**Peer review**	−0.7939297	0.001	−1.25954 ‒ 0.3283196
**Pop based**	−0.5347804	0.046	−1.059428 ‒ 0.0101331
**Sample size**	−0.0000966	0.001	−0.0001561 ‒ 0.0000372
**Rapid test**	−0.5483888	0.066	−1.133172 ‒ 0.0363943
**cons**	−1.181352	0.000	−1.754376 ‒ 0.6083286
viii. There was evidence of bias using the Egger (*p* = 0.000) and Begg (*p* = 0.001) tests.
e) Additional studies:

Additional studies of interest included in this review, as the authors identified at least two surveys in these groups, were in indigenous populations, in immunosuppressed patients (i.e., diagnosed with cancer or undergoing solid organ transplantation), and people who self-requested the test for diagnose SARS-CoV-2 infection, as shown in Table 1. The prevalence of SARS-CoV-2 antibodies in indigenous populations ranged from 60.71% to 83.17%; in immunosuppressed patients, the variation was from 8.17% to 30.70%, and in people who self-requested the test, the variation was from 11.75% to 27.33%.

## Discussion

The authors systematically reviewed seroprevalence studies of SARS-CoV-2 antibodies conducted in Brazil and identified fifty-four studies from all Brazilian states. The present review indicated that the overall seroprevalence of SARS-CoV-2 in Brazil was 11.0% (95% CI 11.0‒12.0) and the heterogeneity among the studies was substantial (99.54%%). In subgroup analyses, the authors observed that the prevalence of SARS-CoV-2 antibodies was 13.0% in blood donors, 9.0% in the population-based surveys, 13% in schoolchildren, and 9.0% in the studies that used the commercial immunochromatographic assays to identify the presence of anti-SARS-CoV-2 antibodies.

As expected, seroprevalence increased over time, from very low figures in the first trimester of 2020, to high proportions in the second half of the year. Seroprevalence in Brazil followed similar trends as observed in other countries, such as Spain, where the prevalence was estimated at 5% after the first epidemic wave [60], and the United States, 3.5% among blood donors [61,62], in July 2020. A large increase in the proportion of infected people during 2020 was also observed, both in developed countries, such as the United States and in developing countries, such as India. In the first, the seroprevalence among blood donors rose to 83.3% in May 2021, when combining natural infection and vaccine-induced seroconversion (Jones et al. 2020). In the latter, seroprevalence in the general population was 0.73% in May‒June and increased to 24.1% in December 2020 [63].

Large differences in seroprevalence were observed amongst the different regions of Brazil. Seroprevalence increased from the South to the Northern region, where the Amazon rainforest is located. This region was particularly hit by the second pandemic wave, being the probable emergence of the gama variant of SARS-CoV-2 in Brazil [64], more transmissible than the previous ones.

The Northern Region of Brazil showed high seroprevalence already in the first seroepidemiological surveys, carried out in the second quarter of 2020. In the first national survey, whose data collection was carried out in May 2020, nine of the ten municipalities with the highest seroprevalence in the country were in this region [16]. In the city of Manaus, one of the largest metropolises in the Amazon region, the seroprevalence among blood donors reached values above 40% in the same period. [24] At the other extreme, the Central-West region had a lower seroprevalence, even considering that this region had a lower number of surveys carried out, compared to other regions. The Northeast region, which includes nine states in the country, ranked second in terms of seroprevalence, followed by the Southeast region. Necessary care when interpreting aggregate seroprevalence estimates is that some regions, and within them, some states, carried out a much larger number of surveys than others, and may be overrepresented in the analysis.

Some surveys pointed out a high seroprevalence among the Indigenous peoples, with the Amazon region being the one that concentrates the largest number of indigenous people in the country. Unfortunately, the number of surveys in which data were stratified by skin color/ethnicity was small, which made it impossible to calculate an estimate of seroprevalence according to this variable.

Part of the differences observed in seroprevalence may be related to the type of assay used in the surveys. In the first months of the pandemic, only immunochromatographic assays were available in Brazil. These assays have lower sensitivity than the enzyme immunoassay and chemiluminescence methods [65], Its sensitivity also depends on the type of sample collected, and its performance is worse in samples collected by finger prick. These assays, with this type of biological sample, were the most used in Brazil during the first months of the pandemic, which may have contributed to the underestimation of seroprevalence, although the Northern Region of Brazil showed high seroprevalence already in the first seroepidemiological surveys.

Although the authors detected considerable heterogeneity and publication bias, we could observe that the available data are robust, even including only surveys with a sample size greater than one hundred or only greater than 500; the same would happen if the authors included only surveys published in peer-reviewed scientific journals, with overlapping confidence intervals of prevalence estimates in these cases.

This is the first systematic review of the seroprevalence of SARS-CoV-2 carried out in Brazil before the implementation of the vaccine in the country, which started in January 2021. It was intended to present this methodology of high robustness in an unprecedented infection in the world, with Brazil presenting a very high disease burden. The study also presented the spread of the infection and in which scenarios the effectiveness of vaccination in the country could be estimated.

## Authors’ contributions

Gerusa M. Figueiredo: Conceptualization; writing original draft; writing review & editing.

Fátima M. Tengan: Conceptualization; writing, original draft; writing review & editing, selected the eligible studies by reading the titles and abstracts, and a list of potentially relevant studies was generated.

Expedito J.A. Luna: Conceptualization; writing original draft; writing review & editing.

Sergio R. Campos: Writing review & editing, selected the eligible studies by reading the titles and abstracts, and a list of potentially relevant studies were generated.

## Conflicts of interest

The authors declare no conflicts of interest.

## Appendix Supplementary materials

Image, application 1

## Footnotes

Supplementary material associated with this article can be found, in the online version, at doi:10.1016/j.clinsp.2023.100233.
