# Supplementary material for: Seroprevalence of SARS-CoV-2 in Brazil: A systematic review and meta-analysis
Source: Clinics (Sao Paulo). 2023 Jun 13;78:100233. doi: 10.1016/j.clinsp.2023.100233 (PMC10261714; doi:10.1016/j.clinsp.2023.100233)

**CLINICS-D-22-00468_ Supplementary Material**

**Supplementary File 1**

**Additional File 1** Search strategies (Held on 07/11/2021).

**Supplementary File 2** Estimated prevalence general.


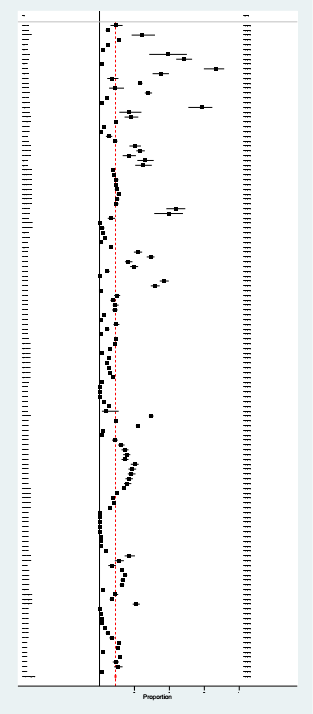


**Supplementary File 3** Estimated prevalence randomly selected samples.


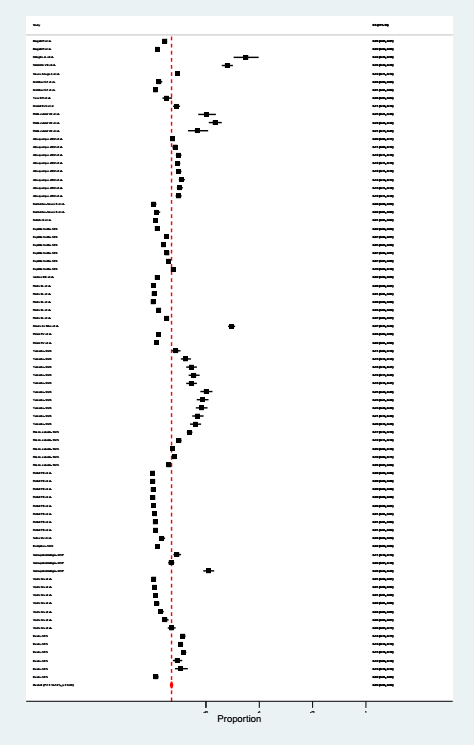


**Supplementary File 4** Estimated prevalence blood donors.


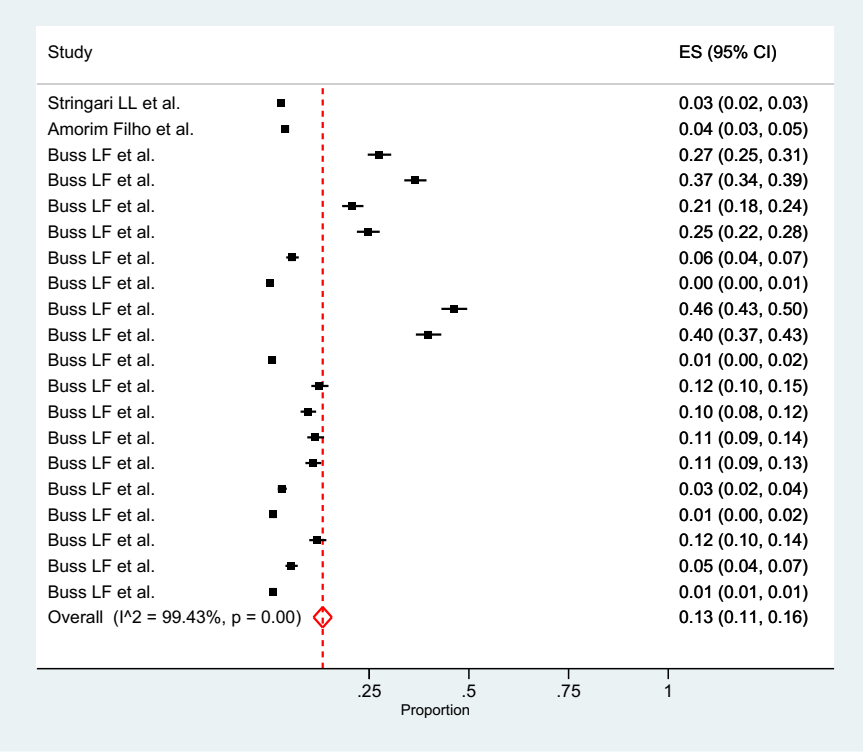


**Supplementary File 5** Estimated prevalence schoolchildren.


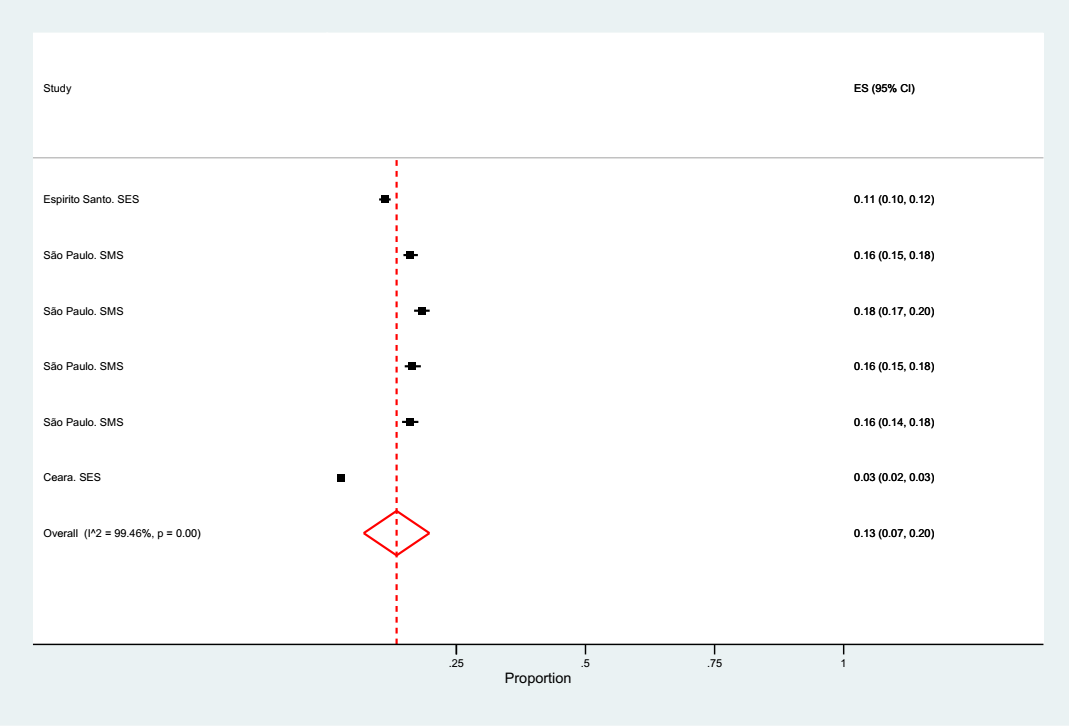


**Supplementary File 6** Estimated prevalence health workers.


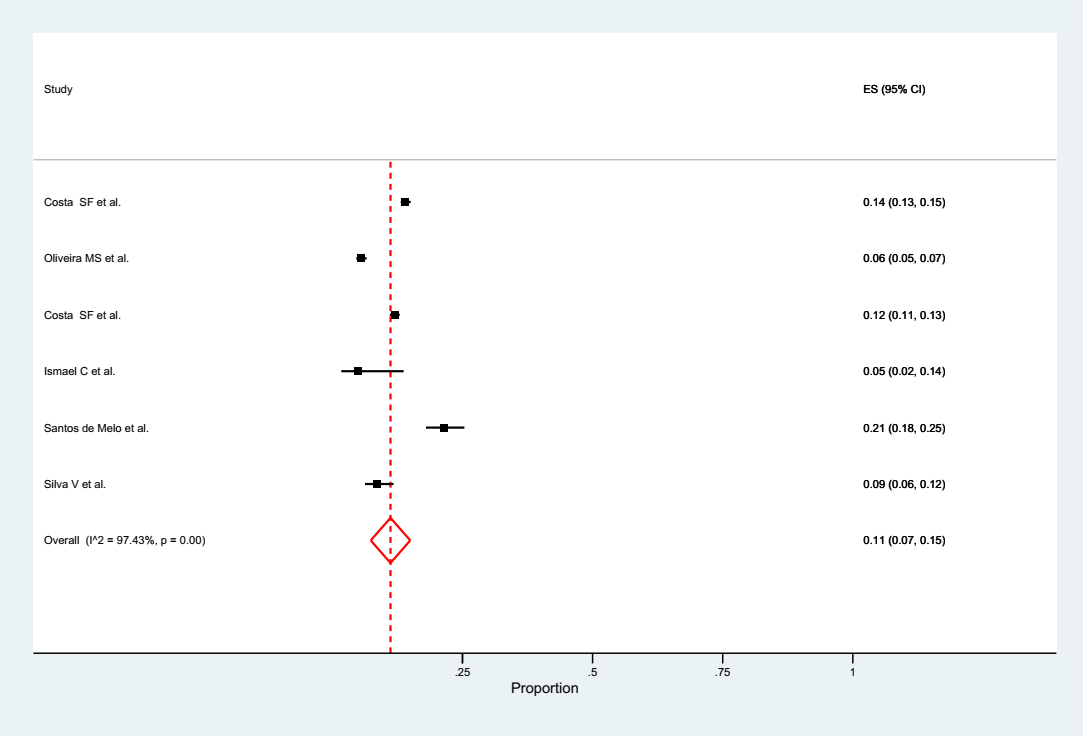


**Supplementary File 7** Estimated prevalence sample size 100.


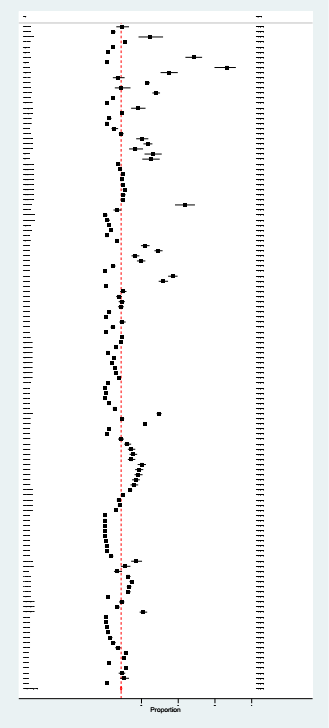


**Supplementary File 8** Estimated prevalence sample size 500.


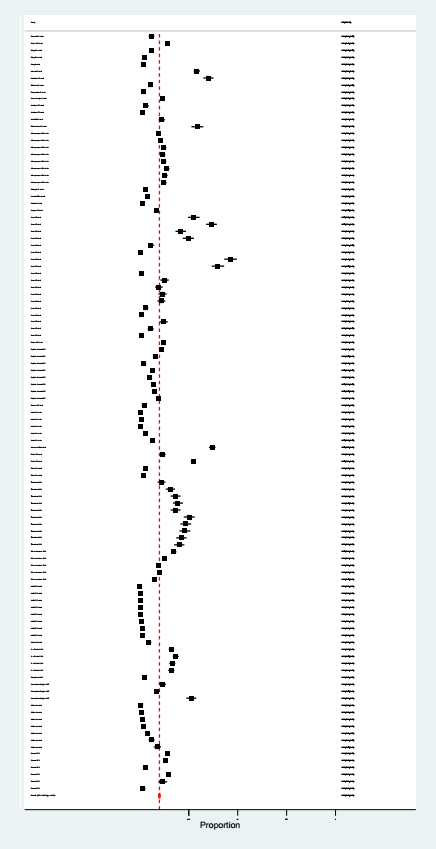


**Supplementary File 9** Estimated prevalence sample size 1000.


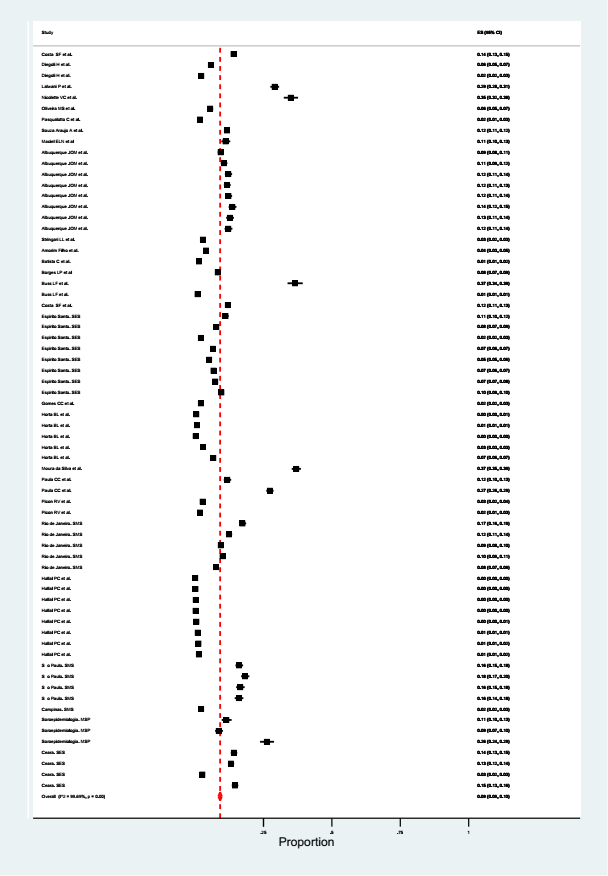


**Supplementary File 10** Estimated prevalence of peer-reviewed.


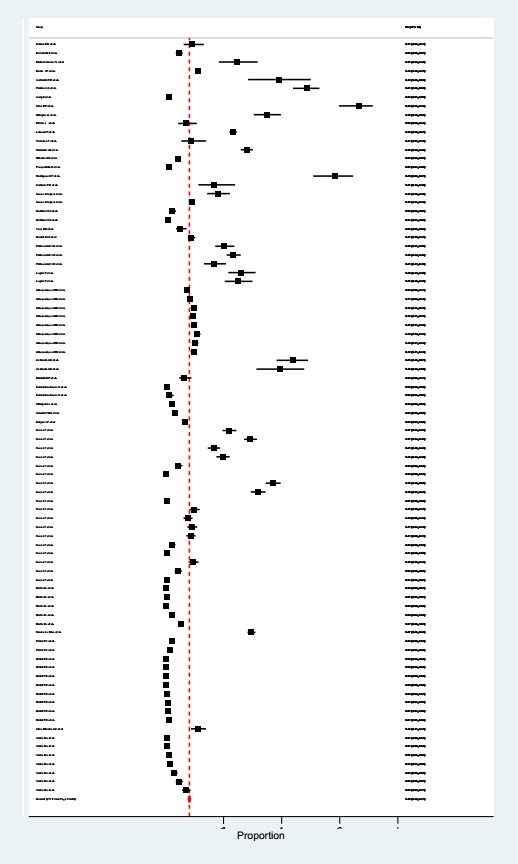


**Supplementary File 11** Estimated prevalence by immunochromatography.


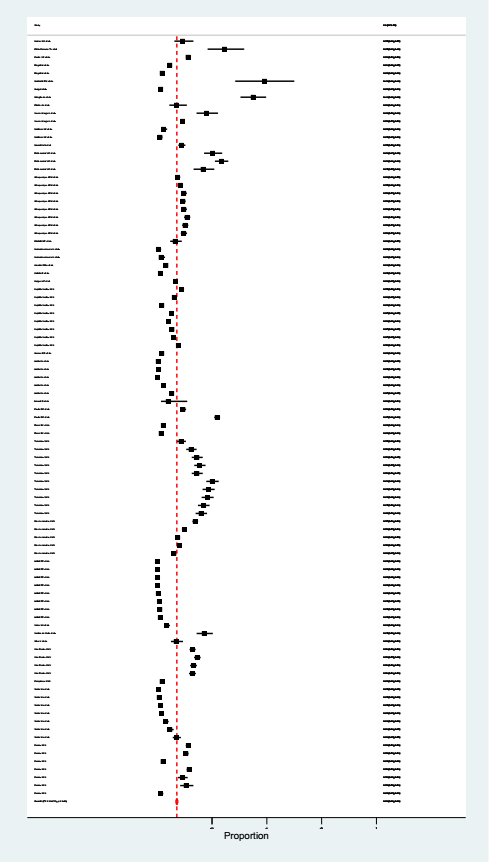

Supplement: Supplementary file 1 [file mmc1.docx]
